# Optimizing the design of population-based patient-derived tumor xenograft studies to better predict clinical response

**DOI:** 10.1242/dmm.036160

**Published:** 2018-10-31

**Authors:** Nicolas Floc'h, Maria Luisa Guerriero, Antonio Ramos-Montoya, Barry R. Davies, Jonathan Cairns, Natasha A. Karp

**Affiliations:** 1 Bioscience, Oncology, Discovery Sciences, IMED Biotech Unit, AstraZeneca, Cambridge CB4 0WG, UK; 2 Quantitative Biology, Discovery Sciences, IMED Biotech Unit, AstraZeneca, Cambridge CB4 0WG, UK

**Keywords:** Patient-derived tumor xenografts, Population studies, Preclinical trial

## Abstract

The high attrition rate of preclinical agents entering oncology clinical trials has been associated with poor understanding of the heterogeneous patient response, arising from limitations in the preclinical pipeline with cancer models. Patient-derived tumor xenograft (PDX) models have been shown to better recapitulate the patient drug response. However, the platform of evidence generated to support clinical development in a drug discovery project typically employs a limited number of models, which may not accurately predict the response at a population level. Population PDX studies, large-scale screens of PDX models, have been proposed as a strategy to model the patient inter-tumor heterogeneity. Here, we present a freely available interactive tool that explores the design of a population PDX study and how it impacts the sensitivity and false-positive rate experienced. We discuss the reflection process needed to optimize the design for the therapeutic landscape being studied and manage the risk of false-negative and false-positive outcomes that the sponsor is willing to take. The tool has been made freely available to allow the optimal design to be determined for each drug-disease area. This will allow researchers to improve their understanding of treatment efficacy in the presence of genetic variability before taking a drug to clinic. In addition, the tool serves to refine the number of animals to be used for population-based PDX studies, ensuring researchers meet their ethical obligation when performing animal research.

## Abstract (precis)

**Summary:** A freely available interactive tool is presented that explores the optimal design of a population PDX preclinical study.

## INTRODUCTION

Oncology is the medical specialty with the highest attrition rate during the development of new therapeutic agents (Kola and Landis, 2004; Grellety et al., 2014). The reasons for the failures of candidate small-molecule drugs have evolved through the years. Indeed, failure due to poor pharmacokinetic profiles have diminished while failures due to efficacy and safety issues have increased (Bunnage, 2011; Hay et al., 2014; Kola and Landis, 2004; Morgan et al., 2018). Numerous solutions have been proposed to tackle the issue of attrition in oncology. As an example, AstraZeneca implemented a major revision of its research and development (R&D) strategy with the aim of improving R&D productivity by establishing a ‘5R framework’ (right target, right tissue, right safety, right patient and right commercial potential) (Cook et al., 2014). The application of the 5R framework is beginning to have an impact, with success rates from candidate drug nomination to Phase 3 completion improving from 4% in 2005–2010 to 19% in 2012–2016 (Morgan et al., 2018). Despite the progress, a major limitation still remains concerning the translational relevance of preclinical *in vivo* models of cancer (Day et al., 2015).

Since the 1950s, when the first report was published on the use of *in vivo* murine leukemia models for the evaluation of drug efficacy (Kirschbaum et al., 1950), efforts have been dedicated to develop preclinical models that better predict the drug response in humans. The development of immunodeficient mice has allowed the engraftment of human tumor cell lines. However, while this approach allows many models to be established with relative ease, these cell-line-derived xenografts (CDXs) bear little resemblance to the original tumors, notably in terms of tumor heterogeneity (Daniel et al., 2009). For this reason, the use of CDXs in evaluating novel agents can play a role in explaining the discrepancy between preclinical efficacy and clinical response in oncology (Sausville and Burger, 2006). The ability to engraft surgically derived tumors from cancer patients has been established for decades, but its systematic use in the drug discovery process has only recently taken off. Patient-derived tumor xenografts (PDXs) retain the tumor mutational profile and the original intra-tumoral heterogeneity (Hidalgo et al., 2014; Tentler et al., 2012). Moreover, mounting evidence suggests that PDXs can better predict an individual's clinical response to therapies (Malaney et al., 2014). However, the majority of studies using PDXs show limited value in predicting potential clinical-trial response at the population level.

In 2011, Bertotti et al. reported for the first time that a cohort of 85 colorectal cancer PDXs responded to cetuximab, an anti-EGFR antibody, with similar rates to those observed in the clinic (Bertotti et al., 2011). Since this first study, interest is mounting in utilizing a clinical Phase-2-like mouse preclinical trial where a number of PDX models capturing the inter-tumor heterogeneity of a human patient population are evaluated. This will allow prediction of the efficacy of a therapeutic agent by assessing the proportion of the population that responds to the proposed treatment. In this type of study, models are classified as responding or not responding based on the ‘modifying response evaluation criteria in solid tumors’ (mRECIST) (Eisenhauer et al., 2009; Gao et al., 2015). While several studies (Gao et al., 2015; Marangoni et al., 2007; Bertotti et al., 2011; Nunes et al., 2015; Ricci et al., 2014; Topp et al., 2014; Zhang et al., 2013) have demonstrated the successful application of a population PDX trial, as yet there is little guidance on the design of such studies. These studies are technically challenging and expensive, which means that there is an equilibrium to be found between sufficient sample size (number of models and number of replicates per model) to capture inter-patient heterogeneity and experimental complexity with associated cost and 3Rs considerations (replacement, reduction, refinement).

In population PDX studies, the primary outcome is an estimation of the proportion of a population responding to the treatment. Within a drug discovery pipeline, this outcome is compared to a go/no-go threshold, which is the decision point on whether to continue or halt further development of this therapeutic agent. This threshold will need to be adjusted based on the disease characteristics, such as the clinical prevalence and the existing therapeutic landscape, to ensure that the new agent will provide a significant benefit for the population of interest while ensuring financial viability of the R&D program. For example, when aiming to position a new drug in the clinic, it is clear that this new drug will have to perform better than the standard of care (SoC), i.e. that the estimated proportion of responders is superior to that seen with the SoC.

In this work, we build an interactive tool to explore the impact of the experimental parameters that need to be defined when designing a preclinical PDX trial to predict the response of a population to a given treatment. We show that the way these preclinical studies are designed can have a strong influence on the reliability of the conclusions obtained. We can minimize the risk of incorrectly progressing with a therapeutic treatment (false-positive result) and minimize the risk of failing to detect that a treatment should progress (false-negative result). The two main experimental parameters requiring consideration are the number of PDX models studied (PDXn) and the number of mice studied per PDX model (PDXr). Other parameters that we include in our model are the biological response rate (Biol_RR), and the classification accuracy (C_Acc). The Biol_RR represents the actual underlying proportion of the population of PDX models that will respond to a treatment of which the experiment is estimating. The C_Acc is the probability of correctly classifying an individual animal from a model as a responder or non-responder based on the mRECIST criteria. This value was defaulted to 0.95 based on a large-scale exploration of PDX behavior by Gao et al. (2015).

By simulating the system with different parameter combinations, we explore the interplay of these factors and their impact on correctly estimating the biological response, looking at both false-positive rate (FPR) and false-negative rate (FNR). As some of the parameters are strongly dependent on the specific drug-disease combination, such as the go/no-go threshold, we provide an easy-to-use interactive tool that enables scientists to explore the interplay of the features they control for the goal of their experiment. We discuss the considerations needed to optimize the design for the therapeutic landscape being studied and the risk of false-negative and false-positive outcomes that the sponsor is willing to accept. This tool will support the effective and efficient use of preclinical PDX models to reduce the high attrition rate of agents entering oncology clinical trials.

## RESULTS

### How does the design impact FPR?

Using simulations, we can assess for false positives (incorrect call to proceed as the estimated proportion responding is greater than the go/no-go threshold) by setting the underlying biological response rate below the go/no-go threshold and asking how many experiments would return a go outcome.

#### How does PDXn impact the FPR?

**Fig. 1.**
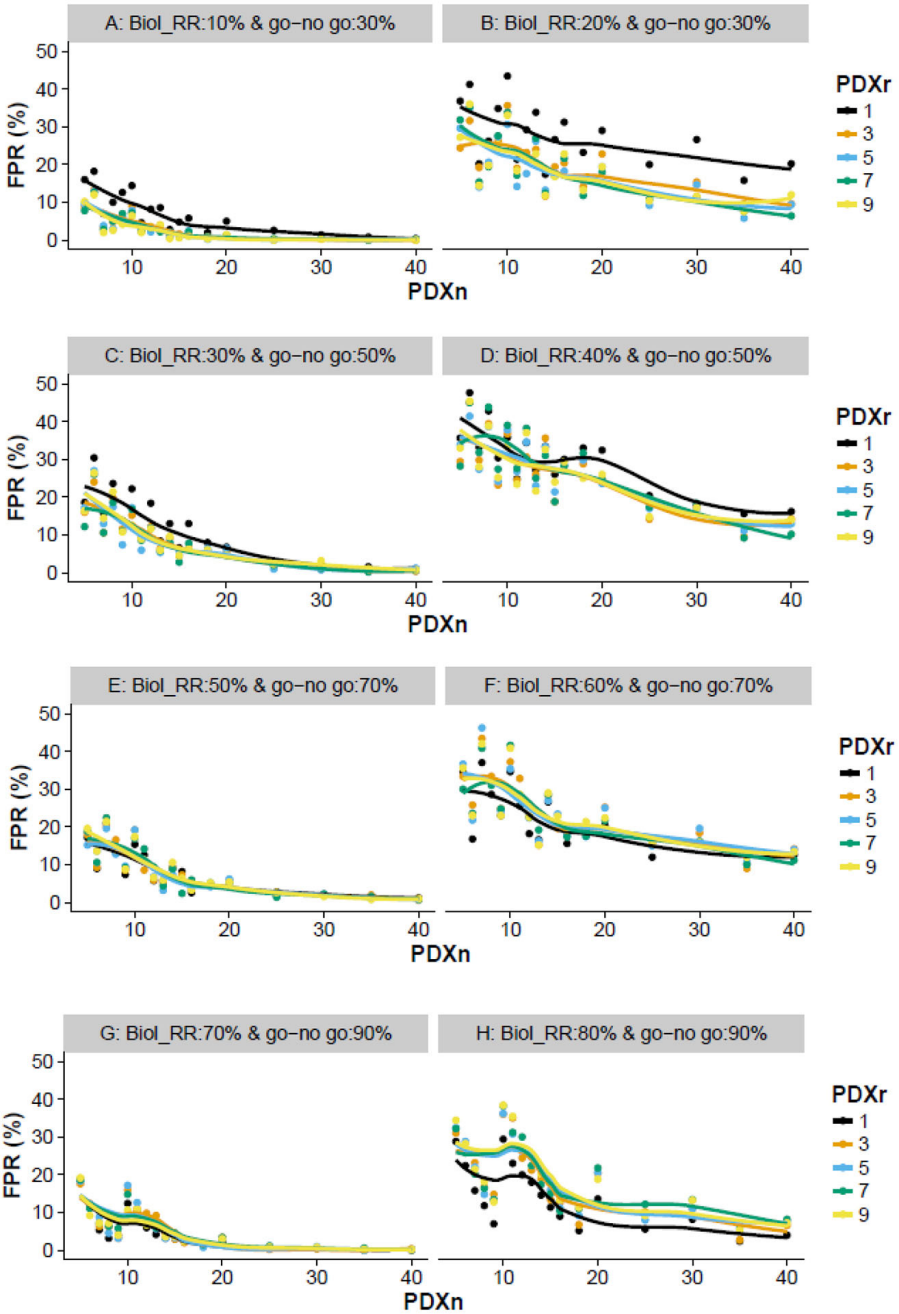
**Exploration of various design** **features that impact the FPR.** For a variety of go/no-go thresholds, the impact of varying PDXn and PDXr on the proportion of false calls (FPR) was explored. (A,B) Visualizes the behavior with a go/no-go threshold of 30% when the Biol_RR was 10 and 20%, respectively. (C,D) Visualizes the behavior with a go/no-go threshold of 50% when the Biol_RR was 30% (C) and Biol_RR was 40% (D). (E,F) Visualizes the behavior with a go/no-go threshold of 70% when the Biol_RR was 50% (E) and Biol_RR was 60% (F). (G,H) Visualizes the behavior with a go/no-go threshold of 90% when the Biol_RR was 70% (G) and Biol_RR was 80% (H). Simulations were used to explore the impact of varying these design features on the proportion of studies where the estimated response rate would have exceeded the go/no-go threshold. For each scenario, 500 simulations were run to enable the average behavior to be assessed.

The exploration of PDXn on the FPR identifies a number of relationships independent of the target go/no-go threshold. Firstly, as PDXn increases, the FPR decreases (Fig. 1). Secondly, the closer the underlying Biol_RR is to the go/no-go threshold, the higher the FPR (Fig. 1); although, it is important to note that the closer the Biol_RR is to the threshold, the less of a concern a false positive would be. Finally, it is worth noting that, if a line was drawn linking each point sequentially rather than a smooth line, this would highlight that the relationship between the FPR and number of models is noisy when the sample size is low (Fig. 1). This relationship arises owing to the discrete nature of the data, in that only whole animals can be classed as responders or non-responders, resulting in the go/no-go threshold implemented varying in its stringency. For example, when the underlying population has a 20% response rate and a go/no-go threshold of 30%, a false-positive outcome will only arise for a study with PDXn=6 when two or more models are responders, which equals an implemented threshold of 33.3%. In comparison, when PDXn=7, a false call can only arise when three or more models are responders, which equals an implemented threshold of 43%.

#### How does PDXr impact the FPR?

The impact of PDXr was found to depend on the go/no-go threshold of interest and the associated Biol_RR. When the go/no-go threshold of interest is low (e.g. <50%), as the PDXr increases from 1 to 3 animals there is a small reduction in the FPR, but thereafter no noticeable improvement (Fig. 1). Increasing PDXr has limited impact on the FPR because of the high classification accuracy and the use of the mode (i.e. the most frequent result) in classifying the model as a responder or non-responder. With the use of the mode, to incorrectly classify a model, multiple rare events have to happen. Considering the scenarios with one animal per model, the high classification accuracy means there is only a 5% chance of the experimental results leading to an incorrect classification of the model, whereas, with three animals per model, the incorrect classification is reduced to 1%.

A counter-intuitive effect was observed as the go/no-go threshold and associated Biol_RR was increased, and arises from the relative proportion of responding and non-responding models and the impact of a misclassification in each of these on the FPR. When non-responders dominate, misclassification leads to an overestimation of the underlying Biol_RR, increasing the FPR, whereas, when responders dominate, misclassification leads to underestimation of the underlying Biol_RR, decreasing the FPR. This imbalanced proportion results in the observation that, as the underlying Biol_RR increases towards 50% and therefore a higher go/no-go threshold is used, the positive effect of increasing PDXr on the FPR diminishes. Thereafter, as the Biol_RR exceeds 50%, increasing PDXr from 1 to 3 leads to an increase in the FPR (Fig. 1).

### How does the design impact FNR?

Within a simulation, we can assess the FNR (sensitivity: the ability to correctly classify a population-drug combination as having a response rate above the go/no-go threshold) by constructing scenarios where the Biol_RR is above the go/no-go threshold and therefore any experiments where the estimated biological response rate is below the thresholds are false-negative calls.

#### How does PDXn and PDXr impact the FNR?

**Fig. 2.**
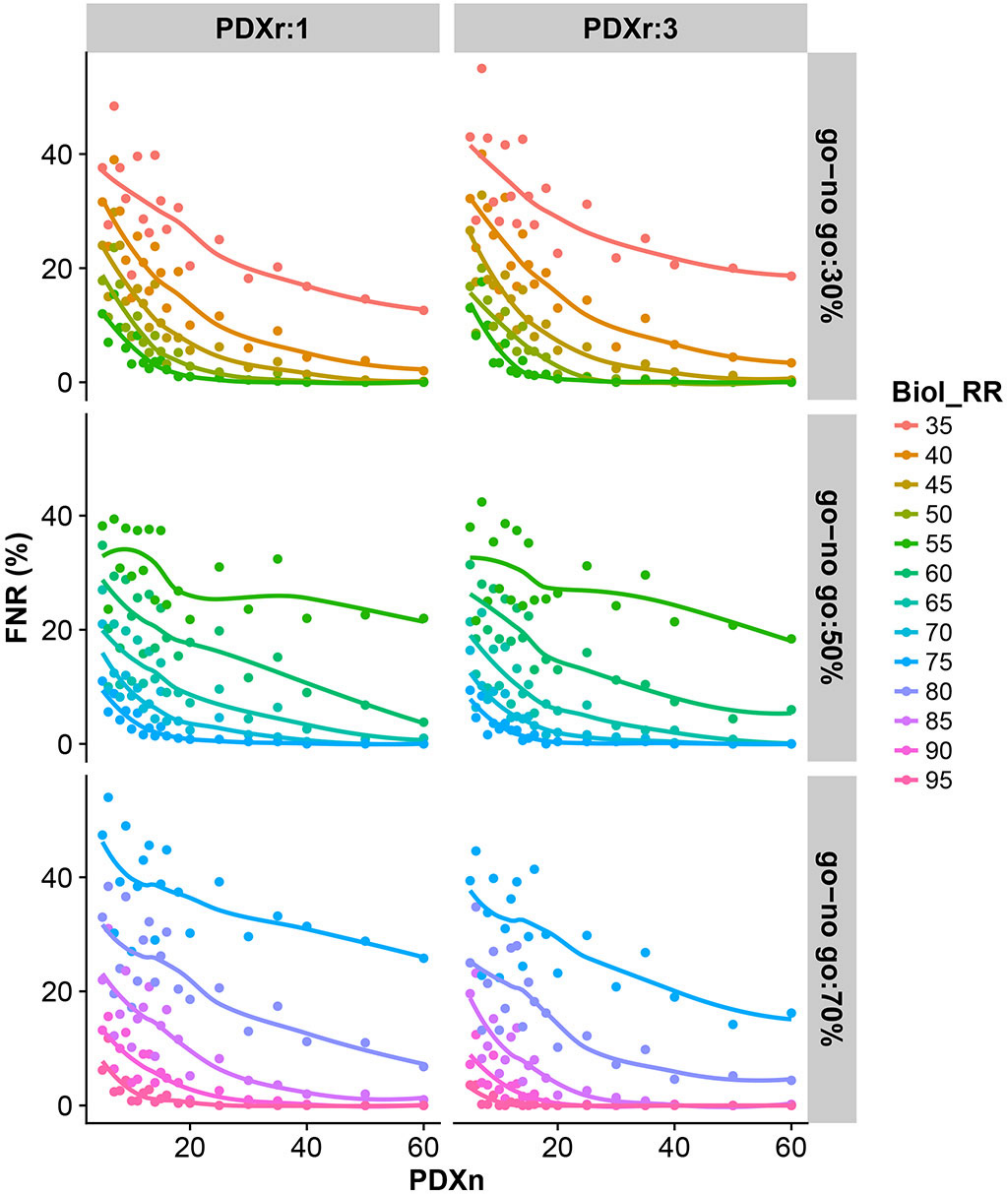
**Impact of PDXn and PDXr on FNR.** Simulations were used to explore the impact of varying PDXn and PDXr on the proportion of studies where the estimated response rate would have failed to exceed the go/no-go threshold (30, 50 or 70%) for a number of Biol_RR. For each scenario, 500 simulations were run to enable the average behavior to be assessed.

The exploration of PDXn and PDXr on the FNR identifies a number of relationships independent of the target go/no-go threshold. Firstly, the higher the Biol_RR for a go/no-go threshold, the lower the FNR as there is more signal in the population and therefore less likelihood for the estimated Biol_RR to be below the go/no-go threshold (Fig. 2). Secondly, increasing PDXn decreases the FNR but this effect has diminishing returns (Fig. 2). Finally, there is no practical difference in sensitivity as the PDXr is increased from 1 to 3 mice per model (Fig. S1). An exception to this was seen when the go/no-go threshold was high (Fig. 2, go/no-go: 70%), where there was up to a 10% decrease in FNR when the PDXr increased from 1 to 3. In these scenarios, the underlying Biol_RR was very high and, with the heterogenous response of most tumors to treatment, an unlikely situation.

**Fig. 3.**
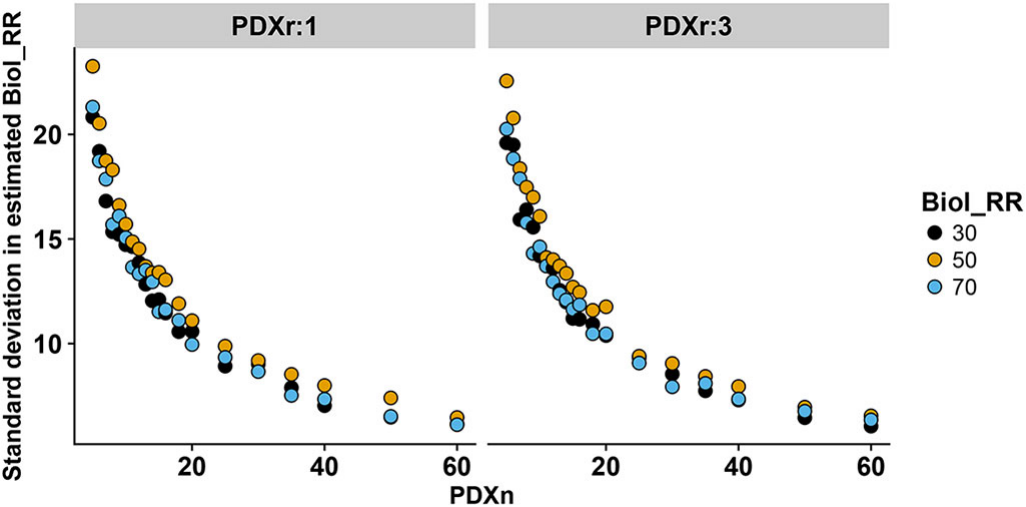
**Impact of PDXn and PDXr on standard deviation in estimated response rate.** The impact of varying PDXn and PDXr on the standard deviation in the estimated response rate was investigated. For each scenario, 500 simulations were run to enable the average behavior to be assessed.

The above observations can be understood by examining the variation in the estimated response rate from the simulations for various designs (Fig. 3). When there is a greater variability, more of the experiments for a scenario will be incorrectly classified as above or below the go/no-go threshold. Supporting the observation that the FNR decreases as PDXn increases, we see the variance decreasing as PDXn increases. Furthermore, we see that PDXr has negligible impact on the variance and hence there is no practical impact of PDXr on the FNR.

### Selecting the optimum design

#### Selecting the PDXn and PDXr based on the evaluation of the FPR

Looking across the various scenarios tested, we cannot make a general recommendation on the actual design that you would select, as the FPR depends not only on the go/no-go threshold of interest but also on the tolerance of false-positive calls the sponsor is willing to tolerate and at what point a false-positive call is alarming (Table 1). Consider a go/no-go threshold of 50%, when the underlying Biol_RR is close (such as 49%), it is very easy for a false-positive call to arise and hence the FPR would be high. However, a FPR in this situation is less concerning. In contrast, we would be very concerned at false-positive calls when the underlying Biol_RR is 20% lower than the expected.

**Table 1.**
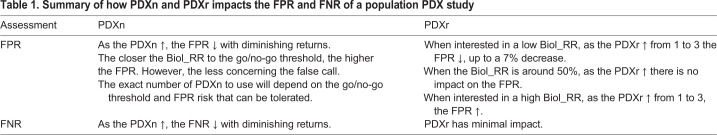
**Summary of how PDXn and PDXr impacts the FPR and FNR of a population PDX study**

We have demonstrated that, for scenarios where the experimenter is interested in a high response rate, there is no value in increasing the number of animals per model. However, when interested in a disease-drug combination predicted to have a low response rate, the FPR can be reduced by increasing the PDXr from 1 to 3 animals per model. For example, for a go/no-go threshold of 30%, using 3 animals rather than a single animal decreases the FPR when the true response rate is 20% by, on average, 7.9% (s.d.=2.52). The tool allows an exploration of the FPR for these scenarios. For example, the reduced risk achieved by increasing the PDXr from 1 to 3 animals allows a cost-benefit analysis to be conducted.

#### Selecting PDXn and PDXr based on the evaluation of the FNR

In terms of the FNR, PDXr had minimal impact and therefore does not need to be considered further (Table 1). Whereas increasing the PDXn decreased the FNR by increasing sensitivity. As the number of models is increased, there is a rapid decrease in the FNR and then we see diminishing returns. The challenge, which is dependent on the operator risk position, is which Biol_RR and go/no-go threshold to focus on to drive the decision and the target FNR to aim for.

## DISCUSSION

In oncology, the project success rates from preclinical research to launch of a new drug is around 4% (Morgan et al., 2018). Improving the predictivity of preclinical models and the way they are currently used will be critical to reduce attrition in drug development pipelines. Preclinical population PDX studies, which mimic a Phase 2 clinical trial, have been proposed as a solution to assess a drug's efficacy in the presence of genetic variability.

Using simulations, we have explored the interplay of how a population PDX study design impacts the FNR and FPR experienced for a variety of go/no-go threshold decision points. Looking across these simulations, we have made some general observations of how changing either the PDXn or PDXr impacts the reliability (FPR) or sensitivity (FNR) of a population-based PDX experiment. However, we cannot make exact recommendations because an experiment will need to be designed for the therapeutic landscape being studied and consider the risk of false-negative and false-positive outcomes that the sponsor is willing to accept. Therefore, we have built and made freely available an easy-to-use tool to explore the impact of these factors for the scenario of interest, supporting the selection of the right design for each situation. (See Resource section of the Materials and Methods for details on accessing this tool.)

This tool makes a number of assumptions. In particular, we assume that the C_Acc is consistent across all models and is accurately estimated. This is a robust assumption, as it was based on the large-scale findings of Gao et al., who studied ∼1000 PDXs with a diverse set of driver mutations (Gao et al., 2015) and is high because of the implementation of mRECIST (responder or non-responder) giving only two classifications with little granularity. The classification of a responder arises from combining tumors that exhibit a complete response, partial response and a stable disease into one ‘responder’ group. Within this classification is a wide range of efficacy. The low *n* population PDX design relies on a high classification accuracy, which has been achieved by using these coarse classifications. Population PDX studies with go/no-go thresholds therefore have a limitation: they only assess the proportion responding and take no account of the size of the response seen. This tool mimics the concept behind a population PDX study and therefore relies on a binary outcome with a known classification accuracy. Potentially, different inhibition thresholds could be used to classify models as responders or non-responders; however, we would need an estimate of the classification accuracy in this scenario to inform the optimum design. We have confidence in the 95% value with the classification used to date as it arose from a study of 2138 single-animal responses from 440 unique treatment models (Gao et al., 2015).

We also assume that the PDXn selected are independent and fully represent the variability within the target population; however, there is a potential risk that, in a disease, some tumors are not represented due to their biological characteristics rendering them harder to grow as PDXs. Implicit in a low *n* population PDX study is the assumption that the PDX models have been correctly classified and therefore represent the target population of interest. This assumption, particularly in the presence of tumor heterogeneity, highlights the importance of validating the model prior to its use within such a study. It should be noted that this tool has been developed to consider the design of population-based PDX studies with the objective of assessing whether the go/no-go threshold has been reached for a compound. If the interest is in comparing proportions of populations that respond to a compound then the experiment design will need to be optimized for this alternate goal and this manuscript does not inform such a scenario.

We have developed this interactive tool to enable the implementation of population-based PDX studies in an effective manner to improve the understanding of how efficacious a treatment is in the presence of genetic variability. We believe that the use of this tool will enable selection of a design that is robust and reproducible, thus improving the prediction of a drug's efficacy. Importantly, this will also have a positive impact on the 3Rs (replacement, reduction, refinement; Russell et al., 1959) as they will ensure the experiments are appropriately designed for the research goals. The data generated from a robust population-based PDX study will better inform the decision-making process on whether to proceed with clinical trials and therefore should reduce the attrition rate in oncology drug development programs.

## MATERIALS AND METHODS

The statistical software R version 3.3.1 (https://www.r-project.org/) was used to construct simulations of PDX population experiments to explore the impact of varying various features of these studies, and the subsequent FPR and sensitivity were assessed. Within each experiment, the following arguments were defined. The C_Acc is the probability of correctly classifying an individual animal from a model as a responder or non-responder based on the mRECIST criteria. This value was defaulted to 0.95 based on the large-scale exploration by Gao et al. (2015). The initial mRECIST criteria classify a response of an animal to a treatment by assessing the tumor volume change with time and classifying the response as complete response, partial response, stable disease or progressive disease. This is converted into a binary classification by collapsing complete response, partial response and stable disease into a responder classification, with those with progressive disease being classed as a non-responder (Gao et al., 2015). The go/no-go threshold is the minimum acceptable proportion of the population that responds to the treatment that is deemed sufficient to proceed the treatment to the next step in the drug development pipeline. The Biol_RR is the true underlying biological response seen in the population being studied and is a feature of the disease and treatment combination. PDXn represents the number of PDX models to be studied and PDXr represents the number of mice studied per PDX model. PDXr is restricted to an odd number as the classification of a PDX model as a responder or non-responder is based on the most frequent outcome. The simulation first mimics the biology: for a given underlying biological response rate and number of models to be studied, a binomial distribution is used to obtain a selection of PDX models with a defined classification (responders or non-responders). Then, the simulation mimics the experiment: each PDX model is classified as a responder or non-responder after using a binomial distribution to mimic the sampling of animals for a model, taking into account the number of animals studied per model and the classification accuracy. The model classification is determined as the mode of the classification received from the animals studied for that model. This process was then repeated 500 times. The input variables were altered to allow an exploration of how they impacted the resulting sensitivity and FPR.

### Resource

A point-and-click easy-to-use interface has been made freely available to explore the interplay of these features on the resulting FPR and sensitivity. The interface has been constructed as a Shiny (Chang et al., 2016) application using R Core Team (2013) and can be downloaded from the CRAN repository. It has been published under the GNU General Public License, which guarantees end-users the freedom to run, study, share and modify the software.

To install and run the interactive tool locally:

(1) Download the latest version of R from www.r-project.org.
(2) Install the populationPDXdesign library and the dependencies by typing in the R console:
  install.packages(“populationPDXdesign”, dependencies=TRUE, repos=‘http://cran.rstudio.com/’).
(3) Load the populationPDXdesign library by typing in the R console: library(populationPDXdesign).
(4) Launch the tool by typing in the R console: populationPDXdesignApp().
(5) On the left-hand side of the interface that opens up, the parameters (Table 2) for the simulations can be set.
(6) Once the parameters are set, from the top of the page select a tab (false positive rate or sensitivity) of interest. This will activate the simulation process and, once completed, a visualization of the results will appear. (Note: To assess the impact of these bespoke settings, a large number of simulations are run prior to the data being summarized with measures of sensitivity and FPR. Consequently, the graphs take time to render. This time can be shortened by minimizing the range or increasing the size of the step you select in your exploration of the factors.)
(7) If you wish to change the parameters, alter the settings on the left-hand side and then press the GO button to initiate a new cycle of simulations.

**Table 2.**
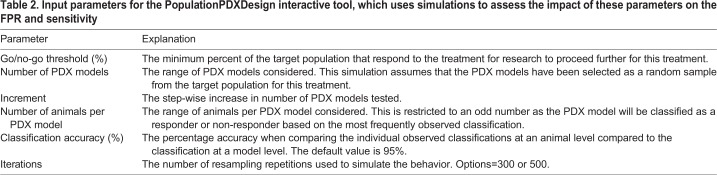
**Input parameters for the PopulationPDXDesign interactive tool, which uses simulations to assess the impact of these parameters on the FPR and sensitivity**

To run the interactive tool through a web browser, follow the instructions below. Note that this resource does have a limitation of number of hours the tool can be accessed in a month. If this situation arises, please wait or follow the instructions above for a local download.

(1) Visit the following site via a web browser: https://mlguerrieroaz.shinyapps.io/populationpdxdesign/.
(2) See steps 6-8 from the local installation on how to use the tool.
